# P-1775. Impact of a Stewardship Intervention Targeting Prolonged Antibiotic Use ( >15 Days) on Antibiotic Utilization, Resistance Patterns, and Costs

**DOI:** 10.1093/ofid/ofae631.1938

**Published:** 2025-01-29

**Authors:** Eunjeong Heo, Yeonju Lee, Hyung-Sook Kim, Ju-Yeun Lee, Seong Jin Choi, Song Mi Moon, Hong Bin Kim, Eu Suk Kim, Kyoung-Ho Song

**Affiliations:** Seoul National University Bundang Hospital, Seongnam, Kyonggi-do, Republic of Korea; Seoul National University Bundang Hospital, Seongnam, Kyonggi-do, Republic of Korea; Seoul National University Bundang Hospital, Seongnam, Kyonggi-do, Republic of Korea; Seoul National University Bundang Hospital, Seongnam, Kyonggi-do, Republic of Korea; Seoul National University Bundang Hospital, Seongnam, Kyonggi-do, Republic of Korea; Seoul National University College of Medicine, Seoungnam-si, Kyonggi-do, Republic of Korea; Seoul National University College of Medicine, Seoungnam-si, Kyonggi-do, Republic of Korea; Seoul National University College of Medicine, Seoungnam-si, Kyonggi-do, Republic of Korea; Seoul National University College of Medicine, Seoungnam-si, Kyonggi-do, Republic of Korea

## Abstract

**Background:**

Inappropriate antibiotic use, particularly prolonged courses, is a significant driver of antibiotic resistance. This study aimed to evaluate the impact of a targeted antimicrobial stewardship program (ASP) intervention on antibiotic utilization, resistance, and costs in patients receiving antibiotics for more than 15 days.

Monthly changes in DOT per 1000 patient-days over the span of 5 years

**Figure:**
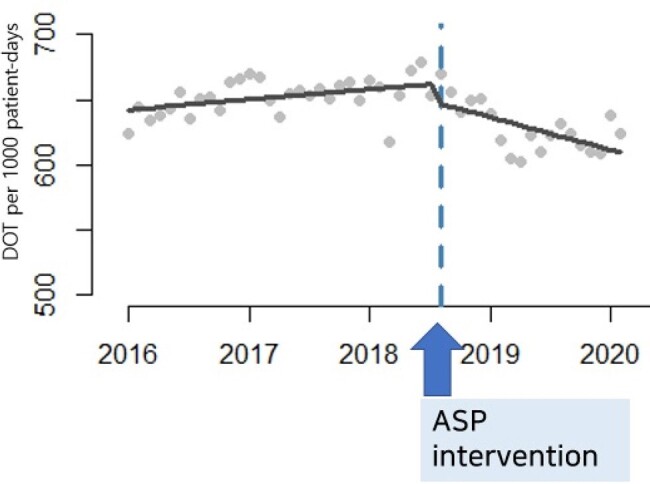

**Methods:**

We employed an intervention targeted adult inpatients receiving antibiotics for more than 15 days since August 2018. An ASP team of pharmacists and infectious disease physicians implemented interventions through direct communication via messengers, visits, and EHR records. The intensive intervention period lasted seven months (August 2018 - February 2019) and continued alongside other stewardship activities afterward. We assessed the changes in antibiotic utilization (days of therapy per 1000 patient-days; DOT, length of therapy per 100 discharges; LOT), length of stay (LOS), resistance rate, and antibiotic costs before (January 2016 - July 2018) and after (August 2018 - January 2020) the intervention, using Interrupted time series analysis, retrospectively.

**Results:**

A total of 193,492 patients were admitted and 587 interventions were conducted over the study period. The intervention resulted in a sustained decrease in DOT (-3.02 DOT; 95% CI: -3.71 to -2.33) and LOT (-1.32 LOT; -1.72 to -0.92). Additionally, a significant reduction was observed in the use of broad-spectrum antibiotics, including third-generation cephalosporins (-0.74 DOT; -1.1 to -0.38) and carbapenems (-0.48 DOT; -0.63 to -0.33). Furthermore, the significant upward trend (0.3% per month; 0.2 to 0.4) in the resistance rate of *E. coli* and *Klebsiella* to third-generation cephalosporins waned (-0.03% per month; 0 to -0.03). Finally, antibiotic costs decreased by $695.9 per 1000 admissions (-889.1 to -502.7). However, no significant change in LOS was associated with the intervention.

**Conclusion:**

This study demonstrates that an ASP intervention focusing on optimizing antibiotic duration can effectively reduce the utilization of broad-spectrum antibiotics, the burden of resistance, and antibiotic costs in a hospital setting.

**Disclosures:**

**All Authors**: No reported disclosures

